# Characterisation of Ovine *KRTAP19-3* and Its Impact on Wool Traits in Chinese Tan Sheep

**DOI:** 10.3390/ani14192772

**Published:** 2024-09-25

**Authors:** Lingrong Bai, Huitong Zhou, Jinzhong Tao, Jon G. H. Hickford

**Affiliations:** 1 International Wool Research Institute, Faculty of Animal Science and Technology, Gansu Agricultural University, Lanzhou 730070, China; lingrong.bai@lincolnuni.ac.nz (L.B.); huitong.zhou@lincoln.ac.nz (H.Z.); tao_jz@nxu.edu.cn (J.T.); 2 Gene-Marker Laboratory, Faculty of Agriculture and Life Sciences, Lincoln University, Lincoln 7647, New Zealand; 3 College of Animal Science and Technology, Ningxia University, Yinchuan 750021, China

**Keywords:** keratin-associated protein, KAP19-3, variation, wool traits, fine wool, heterotypic hair fibres, Chinese Tan sheep

## Abstract

**Simple Summary:**

The curliness of the white wool in Chinese Tan sheep gradually diminishes as the sheep age, but our knowledge of what underpins this change is still limited. To further our understanding, this study characterised the ovine *KRTAP19-3* gene, and the results suggest that variation in the gene affects fibre diameter variability for the heterotypic hair fibres of these sheep.

**Abstract:**

Wool, a natural fibre derived from sheep, can present a challenge to wool processing and manufacturing industries because of the variation in fibre traits. Genetic improvement offers one solution to this challenge, and having a better understanding of the genes that affect wool fibre traits is therefore important. Here, we describe ovine *KRTAP19-3*, a new member of the KAP19 gene family. Phylogenetic analysis revealed its relationship to other known *KRTAP19* gene sequences, and an analysis of the nucleotide sequence variation in *KRTAP19-3* from 288 sheep of a variety of breeds revealed six unique variant sequences. Among these variants, eleven single nucleotide polymorphisms (SNPs) were detected, with six located in the coding region. Three of these coding region SNPs were non-synonymous and would result in amino acid changes. Associations were observed between the presence of specific sequence variants in Chinese Tan sheep and wool trait variation, particularly an increase in fibre diameter variability in the heterotypic hair fibres. These findings enhance our understanding of the genes that encode sheep wool proteins.

## 1. Introduction

The wool from sheep is known for its unique chemical, physical, and biological properties, and it is easily recycled and biodegraded, making it a useful fibre [1]. However, as a natural fibre, variability in wool traits can pose a challenge to the wool processing industry. With many wool traits exhibiting moderate to high heritability [2,3], there is however potential for the genetic improvement of these traits. Accordingly, acquiring a comprehensive understanding of the genes involved in regulating wool fibre characteristics is important. 

Wool fibres primarily consist of wool keratins and keratin-associated proteins (KAPs) [4]. Sheep are reported to possess 17 wool keratins, including 10 type I and 7 type II keratins [5,6,7,8], but the number of KAPs far surpasses this and is anticipated to exceed the 89 KAPs described in humans given the discovery of additional genes encoding the high-glycine/tyrosine (HGT)-KAPs in sheep [9]. This complex genetic landscape, coupled with the polymorphic nature of the keratin genes [10,11,12] and KAP genes (*KRTAPs*) [9,13], along with the potential post-translational modification of the proteins [14], likely contributes to variation in the broad spectrum of wool fibre traits. Many *KRTAPs* in sheep remain unknown [9], highlighting the importance of endeavouring to further identify these *KRTAPs* and investigate their impacts on wool.

The HGT-KAPs are expressed early on in fibre development in the follicles, and this follows the expression of wool keratins [15]. They exhibit differential expression patterns within the cortex, with higher levels of expression observed in the orthocortex, compared to the paracortex [15]. Their abundance varies widely both between and within species, ranging from less than 3% in human hair and wool from the Lincoln breed of sheep, to 4–12% in Merino sheep wool, and up to 30–40% in echidna quills [16]. A reduction in HGT-KAP content has been implicated in the felting lustre mutant observed in Merino sheep [15].

Previously, we have described *KRTAP19-5*, the first member of the KAP19 gene family identified in sheep [17]. The *KRTAP19* family contains seven gene members in humans and is recognised as the largest HGT-KAP group [18]. In this study, we report the identification of another member of the ovine KAP19 gene family.

Additionally, we describe sequence variation in this gene and investigate its influence on wool traits, focusing specifically on Chinese Tan sheep, a breed indigenous to China and renowned for producing wool with a distinctive ‘spring-like’ crimp during the first 35 days after birth [19].

## 2. Materials and Methods

### 2.1. Sheep Investigated and Wool Trait Measurement

This study involved two distinct groups of sheep. The initial group comprised 49 mixed age sheep selected from different farms and comprising seven breeds (Corriedale, New Zealand Romney, Texel, Merino, South Suffolk, Poll Dorset, and Assaf), with seven sheep per breed. This group was exclusively used for screening to ascertain whether variation existed in the gene region targeted and was not subjected to association analyses due to the absence of wool samples and wool trait records. The second group consisted of 239 single-born lambs from the Chinese Tan sheep breed. These were the progeny of 10 sires, with the sire group size ranging from 5 to 46 lambs. There were 114 male lambs and 125 female lambs, with the ewes of these lambs all being from one farm. Fibre samples were gathered from the mid-side area of the lambs at Er-mao (35 days of age). The fine wool fibres and heterotypic hair fibres in the samples were separated based on their differences in length and fibre diameter. The separation involved pressing the base of all the fibres against a flannel board with a solid-edged card, while using the other hand to pull out the longer heterotypic hair fibres.

Measurements of the mean fibre diameter (MFD), fibre diameter standard deviation (FDSD), coefficient of variation in fibre diameter (CVFD), and mean fibre curvature (MFC) were obtained for both the fine and heterotypic fibres. The fine wool samples were assessed by Pastoral Measurements Limited (Timaru, New Zealand), while the hair samples were measured by the New Zealand Wool Testing Authority (NZWTA, Ahuriri, Napier, New Zealand), using the International Wool Textile Organisation (IWTO) standardized internationally ratified testing methods (https://iwto.org/resources/wool-testing-resources/).

Blood samples from individual sheep were collected onto TFN paper (Munktell Filter AB, Falun, Sweden). For DNA extraction, punches of 1.2 mm in diameter were taken from the blood sample and the DNA attached to the TFN paper was purified using a two-step method. This involved incubating the punches in 20 mM NaOH solution for 30 min at room temperature, removal of the NaOH, and a subsequent single wash and equilibration with 1× TE^−1^ buffer (0.1 mM EDTA, 10 mM Tris-HCl, pH 8.0).

### 2.2. PCR Amplification and Single Strand Conformation Polymorphism Analyses

The coding sequence of ovine *KRTAP19-5* was used for a BLAST search of the sheep genome assembly ARS-UI_Ramb_3.0 (GCF_016772045.2) to find similar sequences. Sequences flanking the predicted coding sequence were then used to design the PCR primers: 5′-CTAATACGAGGGCATACATG-3′ (forward) and 5′-TCCAGAATGATCTTTGTTGTC-3′ (reverse), which were then synthesized by Integrated DNA Technologies (Coralville, IA, USA).

Amplification of the targeted region was undertaken in a 15-μL reaction mixture containing a single washed TFN paper punch, 150 μM of each dNTP (Bioline, London, UK), 0.25 μM of each primer, 2.5 mM Mg^2+^, 0.5 U of Taq DNA polymerase (Qiagen, Hilden, Germany), and 1× the reaction buffer supplied with the enzyme and made up to volume with deionized reverse osmosis water. The thermal profile for amplification included an initial denaturation step of 2 min at 94 °C, followed by 35 cycles of 30 s at 94 °C, 30 s at 58 °C, and 30 s at 72 °C. A final extension was then undertaken for 5 min at 72 °C. The temperature cycling was carried out in S1000 thermal cyclers (Bio-Rad, Hercules, CA, USA).

The PCR amplicons generated were analysed using a single-strand conformation polymorphism (SSCP) typing approach. For this, a 0.7-μL aliquot of each was mixed with 7 μL of gel loading dye (98% formamide, 10 mM EDTA, 0.025% bromophenol blue, 0.025% xylene-cyanol). After denaturation at 95 °C for 5 min, the samples were cooled on wet ice and immediately loaded on 16 cm × 18 cm, 14% acrylamide: bisacrylamide (37.5:1; Bio-Rad) gels. Electrophoresis was carried out in Protean II xi cells (Bio-Rad), at 310 volts and 23 °C for 19 h in a 0.5× TBE running buffer. Upon completion of the electrophoresis, the SSCP gels were fixed and stained in a solution containing 10% ethanol, 0.5% acetic acid, and 0.2% silver nitrate for 10 min. The gels were rinsed once with distilled water and then developed with a solution of 3% NaOH and 0.1% HCOH until dark-staining bands appeared against the yellow background. At that point, development was stopped by removing the developing solution and the addition of a solution containing 10% ethanol and 0.5% acetic acid.

### 2.3. DNA Sequencing and Sequence Analysis

PCR amplicons displaying different SSCP banding patterns from sheep that were evidently homozygous in the amplified gene region were sequenced bidirectionally using Sanger sequencing and the original PCR primers at the Lincoln University DNA Sequencing Facility (Lincoln University, Canterbury, New Zealand). Variants detected solely in heterozygous sheep were sequenced using a gel separation-based method explained by Gong et al. [20]. Briefly, a gel slice corresponding to an SSCP band of the variant was cut out from the polyacrylamide gel, macerated, and then used as a template for re-amplification with the original primers. This resulting ‘second’ amplicon was then directly Sanger sequenced. Sequence alignments, translation, and phylogenetic analyses were undertaken using DNAMAN XL (version 10, Lynnon BioSoft, Vaudreuil, QC, Canada).

### 2.4. Statistical Analyses

The statistical analysis were undertaken using Minitab version 16 (Minitab Inc., State College, PA, USA). General linear models (GLMs) were employed to estimate the influence of variation in *KRTAP19-3* on the various wool traits. For this analysis, only genotypes with a frequency greater than 5% were used. To address the issue of undertaking multiple comparisons and reduce the chances of obtaining false positive results, a Bonferroni correction was applied to the genotype comparison models. These models integrated both sire and sex effects, because sire was identified to have an influence on all the wool traits, while sex was identified as a factor impacting certain wool traits. The genotype model was Y_jkl_ = µ + GT_j_ + G_k_ + S_l_ + e_jkl_, where Y_jkl_ is the phenotype value of the jkl^th^ individual, µ is the group raw mean for the trait, GT_j_ is the fixed effect of the j^th^ genotype, G_k_ is the effect of sex, S_l_ is the effect of the l^th^ sire, and e_jkl_ is the random residual effect.

## 3. Results

### 3.1. Identification of Ovine KRTAP19-3

A BLAST search using the ovine *KRTAP19-5* coding sequence (GenBank accession number PQ037232) against the sheep genome assembly ARS-UI_Ramb_3.0 (GCF_016772045.2) revealed two matches in the NC_056054.1 sequence: g.125935545_125935766 (identity = 100%, *E* value = 9 × 10^−113^), and g.125940731_125940952 (identity = 87%, *E* value = 2 × 10^−64^). The first region was ovine *KRTAP19-5*, while the second region contained a sequence that was like ovine *KRTAP19-5*, with this suggesting it was another member of the ovine KAP19 family.

**Figure 1:**
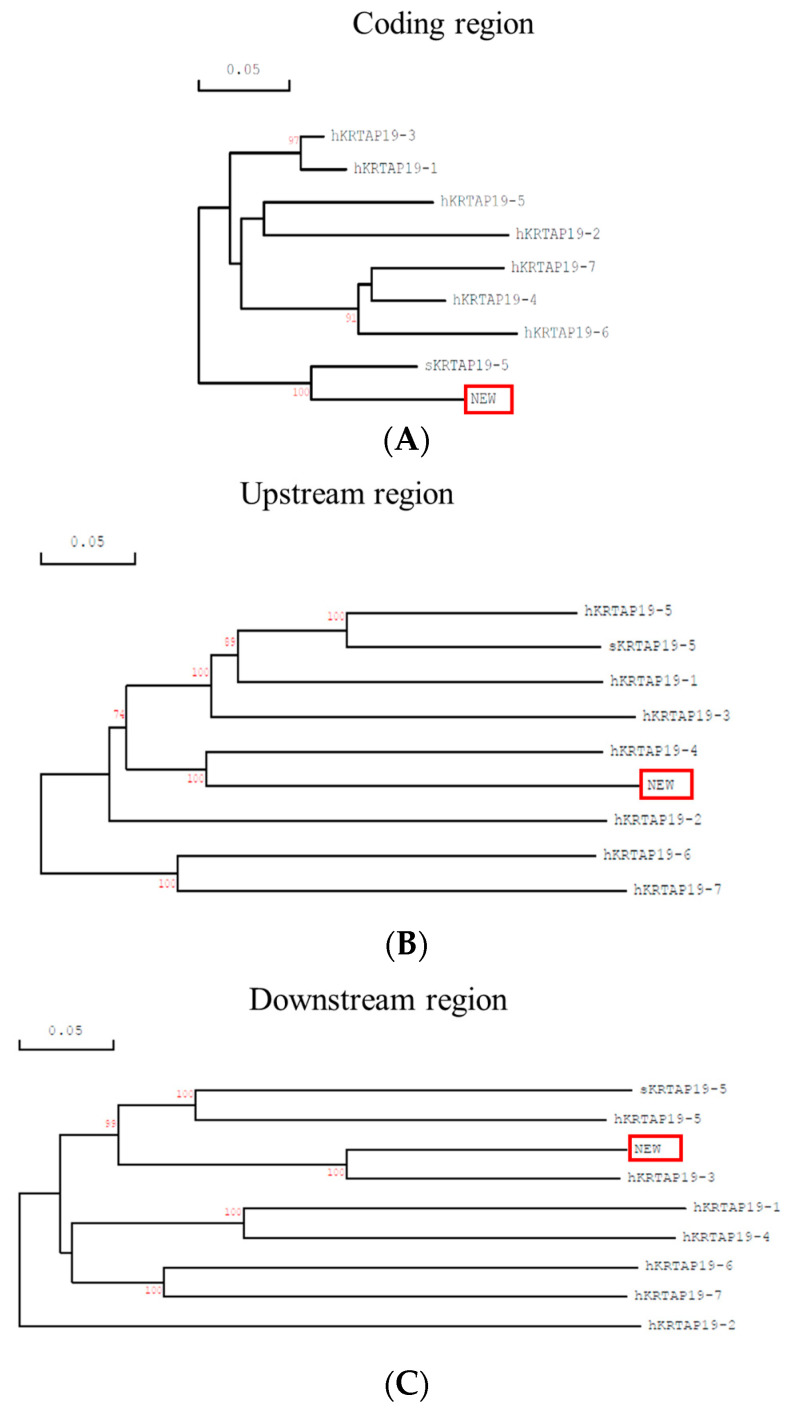
Phylogenetic analyses of the sheep *KRTAP19-n* sequences with members of the human KAP19 gene family. The newly identified sheep *KRTAP19* sequence is highlighted in a box, while the only other sheep sequence is prefixed with ‘s’, and the human genes are prefixed with ‘h’. Analyses are conducted for three different regions: the coding region (**A**), and the 1 kb upstream (**B**) and downstream (**C**) flanking regions. Bootstrap confidence values are indicated at the forks, with only values over 70% shown. The scale bars represent a rate of 0.05 nucleotide substitutions per site.

In a phylogenetic analysis of the coding region, the newly identified sequence and ovine *KRTAP19-5* clustered together, but they were separated from the human *KRTAP19* genes (Figure 1). Interestingly, the newly identified homologous sequence exhibited similarity to human *KRTAP19-4* in the upstream region, and to human *KRTAP19-3* in the downstream region, with the latter displaying shorter branch lengths (Figure 1). This creates a challenge in matching the newly identified sequence to the human ortholog and thus assigning identity. Based on the phylogenetic analysis, it was decided that the newly identified sequence should be designated as ovine *KRTAP19-3*, but it is important to note that while it had some similarity to human *KRTAP19-3*, it may not necessarily be the ortholog.

### 3.2. DNA Sequence Variation in Ovine KRTAP19-3

**Figure 2:**
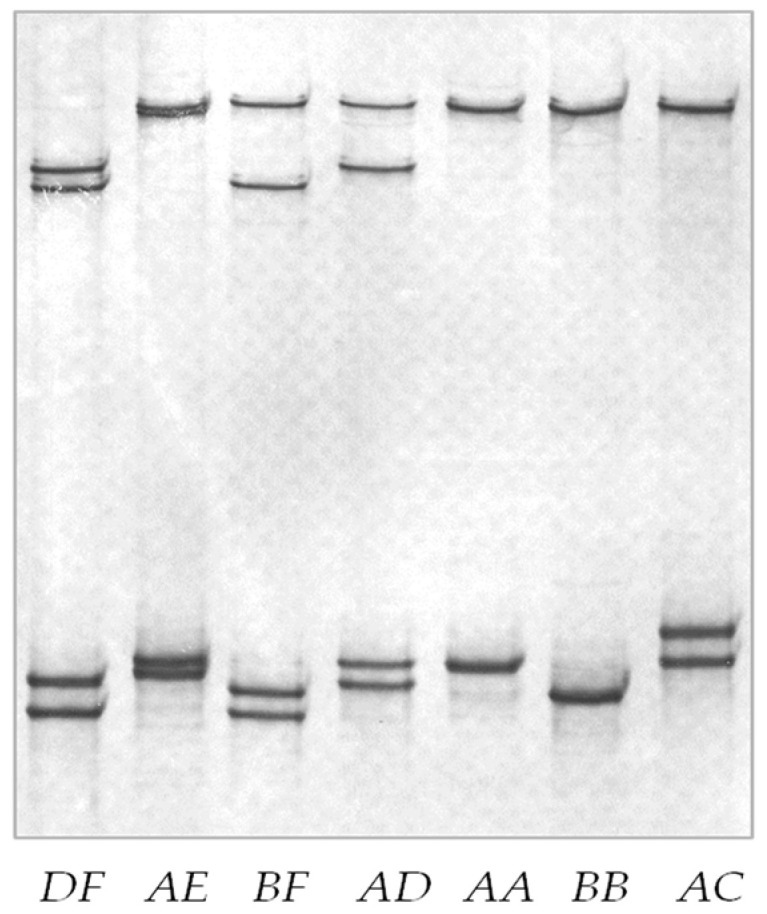
PCR-SSCP gel banding patterns of the ovine *KRTAP19-3* variant sequences. Six different banding patterns (*A* to *F*), corresponding to six different DNA variants are observed in heterozygous forms. The different variants of *KRTAP19-3* are expected to produce different banding patterns on the gels, with each producing two bands that correspond to the two strands of the DNA for any given variant.

**Figure 3:**
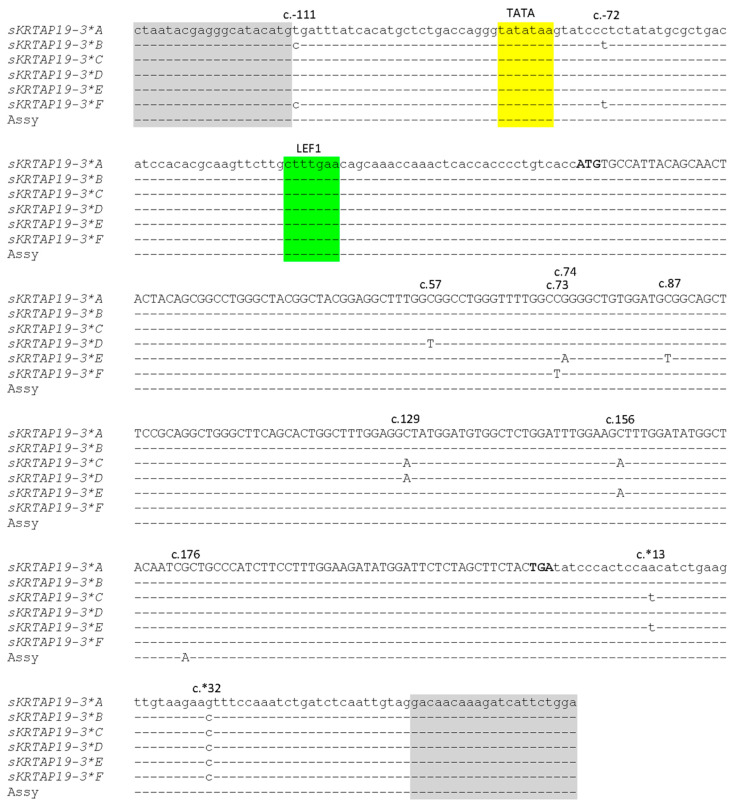
Alignment of the ovine *KRTAP19-3* variant sequences showing the positions of the SNPs identified by Sanger sequencing for the six variants (*A* to *F*). The six variant nucleotide sequences are aligned to the sequence NC_056054.1 (Assy). Nucleotides within the coding region are shown in upper case, while those outside the coding region are in lower case. The start and stop codons are highlighted in bold. The primer-binding regions are shaded in grey, with the putative TATA highlighted in yellow and LEF1 motif in green. The dashes represent nucleotides identical to the top sequence.

Six different banding patterns were detected for the PCR amplicons of *KRTAP19-3* using PCR-SSCP analysis (Figure 2). Sequencing of the representative PCR amplicons revealed six nucleotide sequence variants, with a total of eleven single nucleotide polymorphisms (SNPs) detected among them (c.-111T>C, c.-72C>T, c.57C>T, c.73C>T, c.74G>A, c.87C>T, c.129C>A, c.156C>A, c.176G>A, c.*13A>T, and c.*32G>C). Among these SNPs, seven were in the coding region, two were in the upstream region, and two were in the downstream regions (Figure 3). Of the six coding SNPs, three were non-synonymous, resulting in amino acid changes (c.73C>T, p.Arg25Trp; c.74G>A, p.Arg25Gln; and c.156C>A, p.Ser52Arg). None of the ovine *KRTAP19-3* variants matched the genome assembly sequence precisely, but sequence *A* differed by a single nucleotide from the assembly sequence NC_056054.1. Together, this suggests the potential for further sequence variation in this gene. The frequencies of these variants in the 49 sheep included in the variation screening were 51.0%, 13.3%, 4.1%, 20.4%, and 11.2% for variants *A*, *B*, *C*, *D*, and *F*, respectively.

### 3.3. Associations between KRTAP19-3 Variation and Wool Traits

All six variants of *KRTAP19-3* were detected in the Tan sheep, and 13 genotypes were revealed. Their distribution is summarized in Table 1.

Lambs carrying rare genotypes (with a frequency of less than 5%) were subsequently excluded from the association analyses, as they could potentially cause bias, leaving 211 lambs with the four common genotypes (*AA*, *AB*, *AC*, and *AF*) to be analysed.

For these four genotypes, associations were detected with FDSD and CVFD for the heterotypic hair fibres (Table 2). Lambs with the *AC* genotype had a higher FDSD and CVFD compared to those with the *AA* genotype, suggesting variant *C* is associated with an increase in MFD variation in the heterotypic hair fibres. No associations between *KRTAP19-3* variation and the fibre traits were detected for the fine wool fibres (Table 2).

## 4. Discussion

Here we report the identification of a novel ovine KAP gene that is similar to ovine *KRTAP19-5,* although the number of homologous sequences identified using BLAST searching of the sheep genome assembly NC_056054.1 was lower than might be anticipated, given that seven family members of KAP19 have been described in humans [18]. Only one other sequence like *KRTAP19-5* was detected and, based on its similarity to the human sequences, we have concluded that this sequence would be best classified as *KRTAP19-3*. If other members of the *KRTAP19-n* family do exist in sheep, they would appear to be quite unlike *KRTAP19-5* and currently undetectable using a BLAST searching approach.

If there is greater than expected diversity among the ovine KAP19 members, then that would be supported by the divergence in the evolution of the human KAP19 members (see Figure 1). While some human KAP19 members co-segregate in the dendrograms, others are some distance apart and are more closely related to other KAP families such as human KAP22 and murine KAP18 [18]. This raises the question of whether these human KAP19 members should be placed into one family or if they are from different families. Should they be found, the identification of additional KAP19 gene members in sheep might provide additional insight into this matter.

The 5′ flanking sequence of the novel ovine *KRTAP19-3* sequence contains a DNA binding site for the lymphoid enhancer factor (LEF1), described by the core consensus sequence CTTTG(A/T)(A/T) [21]. Studies involving LEF1 knock-out in mice resulted in the production of hairless individuals [22], while transgenic mice expressing LEF1 (the ligand for the consensus sequence) in the skin epidermis (using a keratin gene promoter) displayed precocious hair growth [21]. This suggests an active role for LEF1 in the development and maintenance of hair or wool follicles. This binding motif (originally designated as the HK1 motif in the promoters of five keratin and KAP genes) [23] has since been found in more keratin and KAP gene sequences from humans, sheep, and mice [21,24,25]. Its widespread presence suggests that it may be an essential characteristic of genes that are associated with hair and wool follicle activity. Typically, this motif is situated 180 to 250 bp upstream from the TATA box [21], albeit with the novel ovine *KRTAP19-3* gene revealed here, and the LEF1 motif appears to be 41 bp downstream of the TATA box (Figure 3).

Analysis of the NC_056054.1 sequence also suggests the presence of an additional LEF1 motif located 343 bp upstream of the TATA box (result not shown), and the novel positioning of the putative LEF1 motif 41 bp downstream of the TATA box in ovine *KRTAP19-3* has not previously reported for any other keratin or KAP gene. As LEF1 mRNA is known to play a central role in hair morphogenesis and gene expression at specific locations and times [21], the unusual location of the LEF1 binding site suggests potential differences in the regulatory mechanisms governing the expression of keratin and KAP genes.

Among the four common genotypes, differences in FDSD and CVFD were detected between the *AA* and *AC* genotypes, but not between *AA* and *AB*, *AA* and *AF*, *AC* and *AB*, or *AC* and *AF*. As all these common genotypes include the *A* variant, a presence/absence model would not be suitable for association analysis in this study. The differences in wool traits between the *AA* and *AC* genotypes suggest that the unique nucleotide changes in variant *C* may be contributing to the phenotypic variation observed in these sheep. Variant *C* differs from variants *A*, *B,* and *F* by three SNPs: c.129C>A, c.156C>A, and c.*13A>T. Among these, SNP c.129C>A is synonymous, c.129C>A is non-synonymous, and c.*13A>T is in the downstream untranslated region. These specific SNPs may have functional effects. Non-synonymous SNPs can alter the protein structure, whereas synonymous SNPs and SNPs in the downstream region can affect mRNA stability and gene expression, and subsequently alter protein structures [26,27].

The association of *KRTAP19-3* variation with changes in FDSD and CVFD, but not with MFD, implies that variation in this gene influences the distribution of heterotypic hair fibre diameter measurements, without affecting their mean size. Considering that some wool fibres can exhibit an elliptical rather than circular cross-section, it is plausible that the gene may affect the ellipticity or morphology of wool fibres, resulting in changes in FDSD and CVFD, but not necessarily MFD. High ellipticity has previously been linked to increased FDSD and CVFD [28]. Alternatively, the variation in *KRTAP19-3* may lead to differences in fibre diameter along its length, from tip to base. This will require further investigation.

The mechanism by which individual genes might affect the ellipticity or morphology of wool fibres is currently unknown. However, wool fibres possess a bilateral structure with orthocortical cells on one side and paracortical cells on the other. These cells differ in their arrangement of intermediate filaments (IFs), the composition of the matrix, and the ratio of IFs to matrix [4]. Variation in ovine *KRTAP19-3* may therefore influence these factors, thereby affecting the morphological structures and mechanical properties of the cells, and consequently impacting the morphology or ellipticity of the fibres.

While the spatial expression of ovine *KRTAP19-3* remains unknown, if it was preferentially expressed in the orthocortical cells, as observed for some HGT-*KRTAPs* [4], its effect may be more pronounced in orthocortical cells compared to paracortical cells, and since fine wool comprises a smaller proportion of orthocortical cells than coarse wool [29], the impact of this gene might be less prominent in fine wool. The absence of any associations being detected with variation in fine wool traits suggests that the effect of ovine *KRTAP19-3* on wool traits is subtle and only becomes apparent when more of this protein is present, as might be expected in the higher MFD in heterotypic hair fibres. Further investigation is warranted to explore whether this is the case.

## 5. Conclusions

The present research identified ovine *KRTAP19-3* and revealed six nucleotide sequence variants of that gene with a total of eleven SNPs. Three of these coding region SNPs were non-synonymous and would result in amino acid changes. Associations were observed between the presence of specific sequence variants in Chinese Tan sheep and wool trait variation, particularly an increase in fibre diameter variability in the heterotypic hair fibres. This work needs to be replicated in other sheep breeds to ascertain whether this association is observed elsewhere, and this may then assist in the development of gene markers for sheep breeding.

## Figures and Tables

**Table 1:** Variant genotype frequencies in the 239 Chinese Tan sheep.

Genotype	Number of Sheep with That Genotype (*n*)	Genotype Frequency
		(%)
*AA*	128	53.6
*AB*	51	21.3
*AC*	15	6.3
*AD*	10	4.2
*AE*	1	0.4
*AF*	17	7.1
*AG*	1	0.4
*BB*	7	2.9
*BC*	4	1.7
*BD*	1	0.4
*BE*	1	0.4
*CF*	1	0.4
*DF*	2	0.8

**Table 2:** Association of common *KRTAP19-3* genotypes with four fibre diameter-related measurements from Chinese Tan sheep.

Fibre Type	Fibre Trait ^1^	Mean ± SE ^2^				*p*
		*AA* (*n* = 128)	*AB* (*n* = 51)	*AC* (*n* = 15)	*AF* (*n* = 17)	
**Fine Wool**	MFD (μm)	16.6 ± 0.20	16.7 ± 0.27	16.6 ± 0.46	16.4 ± 0.46	0.927
	FDSD (μm)	4.2 ± 0.14	4.2 ± 0.20	4.2 ± 0.33	4.1 ± 0.33	0.993
	CVFD (%)	24.8 ± 0.67	25.1 ± 0.92	24.9 ± 1.53	25.0 ± 1.54	0.993
	MFC (°/mm)	65.0 ± 1.31	61.8 ± 1.81	63.6 ± 3.01	64.9 ± 3.02	0.348
**Heterotypic Hairs**	MFD (μm)	29.7 ± 0.39	29.2 ± 0.54	31.1 ± 0.85	29.9 ± 0.88	0.213
	FDSD (μm)	8.1 ± 0.17 ^b^	8.5 ± 0.24 ^ab^	9.4 ± 0.38 ^a^	8.2 ± 0.40 ^ab^	**0.004**
	CVFD (%)	27.3 ± 0.52 ^b^	29.0 ± 0.71 ^ab^	30.2 ± 1.14 ^a^	27.5 ± 1.18 ^ab^	**0.013**
	MFC (°/mm)	46.4 ± 0.85	47.2 ± 1.17	45.8 ± 1.86	46.4 ± 1.93	0.879

^1^ MFD—mean fibre diameter; FDSD—fibre diameter standard deviation; CVFD—coefficient of variation in fibre diameter; MFC—mean fibre curvature. ^2^ Predicted means and standard errors of those means derived from GLMs, with *p* < 0.05 being presented in bold. Means within rows that do not share a superscript letter were different at *p* < 0.05.

## Data Availability

The raw data supporting the conclusions of this article will be made available by the authors on request.
